# Molecular Crosstalk Between SIRT1, Wnt/β-Catenin Signaling, and Inflammatory Pathways in Renal Transplant Rejection: Role of miRNAs, lncRNAs, IL-1, IL-6, and Tubulointerstitial Inflammation

**DOI:** 10.3390/medicina61061073

**Published:** 2025-06-11

**Authors:** Nurhak Aksungur, Murat Kizilkaya, Necip Altundaş, Eda Balkan, Salih Kara, Elif Demirci, Abdullah Uyanik

**Affiliations:** 1 Department of General Surgery, Atatürk University, 25240 Erzurum, Türkiye; dr.aksungur@hotmail.com (N.A.);; 2 Department of Medical Biology, Ağrı İbrahim Çeçen University, 04000 Ağrı, Türkiye; muratkizilkaya25@hotmail.com; 3 Department of Medical Biology, Atatürk University, 25240 Erzurum, Türkiye; 4 Department of Pathology, Atatürk University, 25240 Erzurum, Türkiye; 5 Department of İnternal Medicine, Atatürk University, 25240 Erzurum, Türkiye; auyanik@atauni.edu.tr

**Keywords:** SIRT1, Wnt/β-Catenin signaling, inflammatory, rejection, miRNAs, lncRNAs, IL-1, IL-6

## Abstract

*Background/Objectives*: This study aimed to evaluate the relationship between sirtuin family members (SIRT1, SIRT3, and SIRT6) and Wnt/β-catenin pathways with inflammation during the rejection process following kidney transplantation, as well as to explore their potential roles as candidate biomarkers. *Materials and Methods*: Blood samples were collected from 35 kidney transplant rejection patients and 30 healthy controls. The gene expression levels of SIRT1, SIRT3, SIRT6, and Wnt/β-catenin pathway components were measured using real-time PCR, and miRNA and lncRNA expression levels were analyzed. Statistical analyses were performed using SPSS version 23. *Results*: Significant alterations in SIRT1, SIRT3, and SIRT6 expression levels were observed in rejection patients, suggesting their potential role in disease pathogenesis and as therapeutic biomarkers. Key altered genes included hsa-miR-34c-1, hsa-miR-122b-5b, MALAT1, HOTAIR, LINC00473, TUG, PVT1, SIRT1, SIRT3, SIRT6, WNT1, TCF-LEF, LRP, AXIN1, IL1B, IL6, and IFNB1, all showing significant changes. However, no significant differences were found for miRNAs such as hsa-miR-21-2, hsa-miR-155-5p, and hsa-miR-200b-3p. SIRT1 expression was significantly decreased in the cellular rejection group, with a more pronounced reduction in these patients. Significant differences in SIRT1 expression were observed with interstitial inflammation and glomerulitis. Increased inflammation severity correlated with decreased SIRT1 and increased TCF-LEF, TUG, and miR-21 levels, while tubulitis severity was associated with elevated TCF-LEF and miR-155 expression. *Conclusions*: Along with the activation of Wnt/β-catenin pathways and increased levels of certain miRNAs and long non-coding RNAs (lncRNAs) associated with TCF-LEF transcription factors, the observed decrease in SIRT1 expression may be related to the severity of inflammation and tubulitis. These findings suggest that SIRT1, Wnt/β-catenin pathways, and non-coding RNAs play a role in the rejection process following kidney transplantation and could be considered as potential biomarkers or therapeutic target candidates for future research.

## 1. Introduction

Kidney rejection occurs when, after transplantation, the immune system recognizes the graft as foreign and mounts an immune response characterized by inflammation and tissue damage. This process involves both humoral and cellular immunity: T cells primarily mediate the cellular response, while B cells and antibodies drive the humoral responses [1]. Key mechanisms underlying rejection include MHC incompatibility, donor-specific antibodies (DSAs), and T cell activation through MHC class I and II molecules, triggering the release of cytokines such as TNF-α, IL-6, and IL-1β [2]. These cytokines initiate fibrogenic and inflammatory pathways, contributing to graft injury and loss [3].

At the molecular level, the Wnt/β-catenin signaling pathway regulates the activation and differentiation of key immune cells involved in rejection, including CD4+ and CD8+ T lymphocytes, macrophages, and dendritic cells. This pathway promotes the differentiation of T cells into pro-inflammatory Th1 and Th17 subsets, directs macrophages toward the inflammatory M1 phenotype, and enhances dendritic cell antigen presentation, thereby sustaining inflammation and tissue damage in the graft.

Sirtuins—particularly SIRT1, SIRT3, and SIRT6—modulate rejection by inhibiting the NF-κB signaling pathway to reduce inflammation, controlling the release of pro-inflammatory cytokines, supporting mitochondrial function, and maintaining genomic stability to protect tissues from damage during rejection.

Additionally, long non-coding RNAs (lncRNAs) and microRNAs (miRNAs) form complex regulatory networks that fine-tune gene expression in immune cells. They regulate cytokine production and cellular differentiation, significantly contributing to the progression and severity of graft rejection.

Regulatory molecules including lncRNAs, miRNAs, and inflammatory cytokines—create complex interaction networks influencing gene expression and immune responses, thus contributing to the progression and severity of graft rejection [4,5]. However, limited studies have examined the interplay between sirtuins and Wnt signaling in kidney rejection, and the molecular roles of lncRNAs and miRNAs remain underexplored.

Sirtuins are NAD⁺-dependent deacetylases that are critical for the regulation of cellular homeostasis and inflammation. During kidney rejection, SIRT1, SIRT3, and SIRT6 reduce inflammation and oxidative stress, helping to protect tissues and promote recovery [6]. SIRT1 suppresses inflammatory pathways such as NF-κB and p53, modulates immune cell activity, and controls pro-inflammatory cytokine release, thereby mitigating the severity of rejection [7]. SIRT3 preserves mitochondrial function and limits oxidative stress, which are essential for kidney tissue integrity [8]. SIRT6 maintains genomic stability and curbs pro-inflammatory responses, aiding in post-transplant recovery [9].

Wnt/β-catenin signaling is pivotal in cellular proliferation, differentiation, and immune activation. Overactivation promotes inflammation and fibrosis, worsening graft injury [10]. Pharmacologically targeting this pathway shows promise for reducing inflammation and fibrosis to improve graft survival.

Despite limited direct evidence in kidney rejection cases, studies of sirtuins and Wnt signaling in kidney disease support their involvement in modulating inflammation and tissue damage [7,11]. Further research on their interactions could reveal novel therapeutic targets.

LncRNAs (non-coding RNAs > 200 nucleotides) and miRNAs (~20–24 nucleotides) regulate gene expression at the transcriptional and post-transcriptional levels, influencing cellular processes such as growth, differentiation, and stress response. Their crosstalk fine-tunes immune responses and disease progression [12].

This study aims to elucidate the roles of sirtuins, Wnt/β-catenin signaling, inflammation, and regulatory RNAs in kidney rejection, correlating molecular findings with clinical and histopathological data. Understanding these mechanisms may facilitate the development of novel biomarkers and therapeutic strategies to improve transplant outcomes.

## 2. Materials and Methods

### 2.1. Study Scope and Design

This prospective study was conducted in collaboration with the Departments of Organ Transplantation, Nephrology, Medical Biology, and Pathology at the Atatürk University Faculty of Medicine. The study included 35 patients who developed organ rejection after kidney transplantation and 30 healthy controls.

Kidney transplant patients who developed cellular or humoral acute rejection within the first six months were included in the study. The diagnosis of organ rejection was confirmed using clinical findings and laboratory tests, and patients with rejection were selected.

### 2.2. Materials

#### Study Population and Sample Collection

The study population consisted of patients who developed organ rejection at different stages after kidney transplantation. Thus, the demographic data (age, gender, and type of transplantation) and clinical information of 35 patients diagnosed with organ rejection were recorded in detail.

Blood samples were collected from patients who developed organ rejection immediately after the rejection was detected. These samples were collected according to standard protocols and carefully stored to obtain accurate results in subsequent analyses.

The control group consisted of 30 healthy individuals, selected based on criteria ensuring comparability in terms of graft function, overall health status, and other potential confounding variables. These inclusion criteria were established to enhance the reliability and validity of the obtained data.

Patients included in the study were selected from kidney transplant recipients who developed acute rejection, confirmed by clinical, laboratory, and histopathological findings. Only those with available blood and biopsy samples were included in the analysis.

Patients were excluded if they had no biopsy data, had experienced graft loss, had died, had incomplete clinical records regarding infections or systemic inflammatory conditions, or were under active immunosuppressive therapy at the time of sampling. Immunosuppressive therapy was an exclusion criterion due to its effects on gene expression and inflammatory signaling, which can interfere with the interpretation of molecular data related to the rejection process.

These criteria were defined to minimize confounding variables and to ensure a more accurate evaluation of the molecular alterations specifically associated with the rejection itself.

### 2.3. Ethical Approval

This study was conducted in accordance with the Declaration of Helsinki and was approved by the Clinical Research Ethics Committee of Atatürk University Faculty of Medicine (Approval Code: B30.2 ATA-0.01.00/241, Approval Date: 2025). Written informed consent was obtained from all participants.

### 2.4. Methods

#### Rejection Development and Biopsy Evaluation Process

The clinical and laboratory parameters of the patients were prospectively recorded. Kidney biopsies were performed for patients suspected of rejection, and biopsy results were evaluated. In acute cellular rejection cases, significant inflammation and cellular infiltration were observed in the tubular interstitial areas, while in humoral rejection cases, vascular lesions and findings based on antibody presence were detected. These findings provided valuable information for guiding the treatment process and understanding the severity of organ rejection. Biopsy results were used to assess patients’ responses to treatment, playing a critical role in decisions related to preserving kidney function.

### 2.5. Molecular Analyses

#### 2.5.1. Real-Time PCR Analysis: Examination of Gene, LncRNA, and miRNA Expression Levels

The expression levels of sirtuins (SIRT1, SIRT3, and SIRT6), WNT (WNT, TCF-LEF, LRP, and AXIN1), lncRNAs (MALAT1, HOTAIR, LINC00473, PVT1, and TUG), and cytokines (IL-1B, IL-6, and IFNB1) were determined using real-time PCR (qPCR). RNA isolation was performed using the QIAGEN RNeasy (Hilden, Germany) Plus Mini Kit (Cat. No. 172045918). In this process, 1 mL blood samples from patients and control groups were mixed with 5 mL of EL buffer and incubated on ice for 15 min. After centrifugation, RLT buffer was added to dissolve the samples. These samples were then loaded into purple-colored spin columns in 700 µL volumes and purified through sequential washing steps. High-quality RNA was obtained while maintaining its integrity.

For cDNA synthesis, the QIAGEN RT^2^ First Strand Kit was used. In this stage, a 4 µL RNA sample was mixed with 1 µL of GE buffer and incubated at 42 °C for 5 min. Then, 2 µL of 5× BC3 buffer, 0.5 µL of Control P2, 1 µL of reverse transcriptase enzyme, and 1.5 µL of RNase-free water were added to initiate the cDNA synthesis reaction. This reaction was performed at 42 °C for 15 min and at 95 °C for 5 min, efficiently converting RNA into cDNA.

Real-time PCR amplification was performed using the QIAGEN QuantiTect SYBR Green PCR Master Mix (Cat. No. 208056). For each reaction, 6.5 µL of SYBR Green PCR Master Mix, 0.5 µL of primer, and 5 µL of cDNA were added to PCR tubes and placed in the Qiagen Rotor-Gene qPCR machine. The PCR cycles started with a 15-min denaturation step at 95 °C, followed by 15 s at 95 °C, 30 s of annealing at 60 °C, and 30 s of extension at 72 °C for each cycle. After amplification, a melting curve analysis was performed between 65 °C and 95 °C.

Data analysis was performed by calculating the gene expression levels using the ΔCT (delta cycle threshold) method. ΔCT values were compared between the patient and control groups, and gene expression was normalized using the GAPDH reference gene. Technical replicates were used for each min sample to assess the reliability of the results.

The primers and kits used in the study were provided by QIAGEN. Specific primers designed for the SIRT-1, SIRT-3, and SIRT-6 genes (ID No.: QT01886675, QT00091490, and QT00056812) were used. Additionally, all kits and reagents used during PCR were sourced from QIAGEN, including the QuantiFast SYBR Green PCR Kit (Cat. No. 208056) and RT^2^ First Strand PCR Kit (Cat. No. 77203139), ensuring high specificity and yield for each gene.

#### 2.5.2. miRNA Expression Levels Determination

miRNA Isolation: miRNA isolation was performed using the Qiagen miRNeasy Serum/Plasma Kit (Cat. No.: 74106), according to the manufacturer’s protocol. The following steps were followed: (a) 200 µL of plasma was mixed with 1 mL of Qazol and incubated at room temperature for 10 min; (b) 2 µL of CR RNA and 200 µL of chloroform were added, followed by vortexing for 15 s and centrifugation at 12,000× *g* for 15 min; (c) after centrifugation, the transparent upper layer was carefully transferred to a new tube, and 1.5 times the collected volume of ethanol was added and vortexed; (d) the mixture was transferred in two parts to the RNeasy MiniElute spin column and centrifuged at 8000× *g* for 15 s; (e) the column was transferred to a new collection tube, and 700 µL of RWT Buffer was added and centrifuged at 8000× *g* for 15 s; (f) the column was transferred to another new collection tube, and 500 µL of RPE Buffer was added, followed by centrifugation at 8000× *g* for 15 s; (g) the column was transferred to another collection tube, and 500 µL of 80% ethanol was added and centrifuged at 8000× *g* for 15 s; (h) finally, 14 µL of RNase-free water was added to the column, and the mixture was centrifuged at maximum speed for 1 min to obtain miRNA.

cDNA Synthesis: cDNA synthesis was performed using the Qiagen miRCURY LNA RT Kit (Cat. No. 339340). For each patient, 2 µL of 5x miRCURY SYBR Green mix, 1 µL of 10x miRCURY RT enzyme, and 7 µL of RNA sample were mixed and placed in a PCR machine set at 42 °C for 60 min and 95 °C for 5 min.

Real-Time PCR Amplification: The obtained cDNAs were amplified using the Qiagen miRCURY LNA RT Kit (Cat. No. 339340). For each patient, 5 µL of 2x miRCURY SYBR Green master mix, 1 µL of primer, 1 µL of RNase-free water, and 3 µL of cDNA were added to PCR tubes and placed in the Qiagen Rotor-Gene machine.

PCR Cycle Conditions: The PCR conditions were optimized to ensure accurate amplification and minimize primer–dimer formation:

- Initial denaturation: 95 °C for 2 min to activate the polymerase.
- Amplification cycles: 40 cycles.
- Denaturation: 95 °C for 10 s.
- Annealing and extension: 56 °C for 60 s.

After amplification, a melting curve analysis was performed between 65 °C and 95 °C, with 0.5 °C increments and a 5-s duration for each step.

#### Statistical Methods

Data were analyzed using IBM SPSS V23 and the R programming language (version 4.4.1). Normality was assessed using the Shapiro–Wilk test. For comparisons of categorical variables between groups, Yates’ correction was used. For comparisons of normally distributed variables between two groups, the independent samples *t*-test was used, and for non-normally distributed variables, the Mann–Whitney U test was applied. For comparisons of normally distributed variables across three or more groups, One-Way Analysis of Variance (ANOVA) was used, with post hoc comparisons conducted using the Duncan test. For non-normally distributed variables across three or more groups, the Kruskal–Wallis H test was used, followed by a post hoc Dunn’s test.

The Pearson correlation coefficient was used to examine relationships between normally distributed variables, and Spearman’s rho correlation coefficient was used for non-normally distributed variables. ROC (Receiver Operating Characteristic) analysis was performed to determine cutoff values to distinguish disease. Binary logistic regression analysis was used to identify independent risk factors for humoral rejection, and the Backward Wald method was applied to include independent variables in the model.

To account for the increased risk of false positives due to multiple comparisons, False Discovery Rate (FDR) correction was applied where appropriate.

The results are presented as frequencies (percentages) for categorical variables and as mean ± standard deviation or median (minimum–maximum) for continuous variables. A *p*-value of <0.05 was considered statistically significant.

## 3. Findings

The gender distribution across the groups did not show a statistically significant difference (*p* = 0.150). In the control group, the percentage of males was 51.5%, while in the patient group, it was 71.4%. The percentage of females in the control group was 48.5%, while in the patient group it was 28.6% (Table 1).

Various miRNA, lncRNA, and mRNA expression levels in the patient and control groups were compared, and significant differences between them were observed. The expression levels of SNORD61, hsa-miR-34c-1, hsa-miR-122b-5b, MALAT1, HOTAIR, LINC00473, TUG, PVT1, GAPDH2, SIRT1, SIRT3, SIRT6, WNT1, TCF-LEF, LRP, AXIN1, IL1B, IL6, and IFNB1 were significantly altered in the patient group (*p* < 0.05). These differences suggest that these genes may play a role in disease pathogenesis. However, no statistically significant difference was found between the two groups for hsa-miR-21-2, hsa-miR-155-5p, hsa-miR-200b-3p, and GAPDH levels (*p* > 0.05). Additionally, the mean age of the patient group was higher than that of the control group, and this difference was statistically significant (*p* = 0.006) (Table 2).

A weak positive correlation was observed between hsa-miR-155-5p and SIRT1 (*p* = 0.034), with an FDR-adjusted *p*-value of 0.045, indicating statistical significance. Similarly, a significant positive correlation was found between hsa-miR-200b-3p and SIRT1 (*p* = 0.027; FDR = 0.038), while HOTAIR demonstrated a significant negative correlation with SIRT1 (*p* = 0.014; FDR = 0.025). Furthermore, the association between SIRT1 and TCF-LEF remained significant after FDR correction (*p* = 0.018; FDR = 0.029). Additionally, a significant negative correlation was observed between TCF-LEF and SIRT3 (*p* = 0.016; FDR = 0.027).

**Figure 1:**
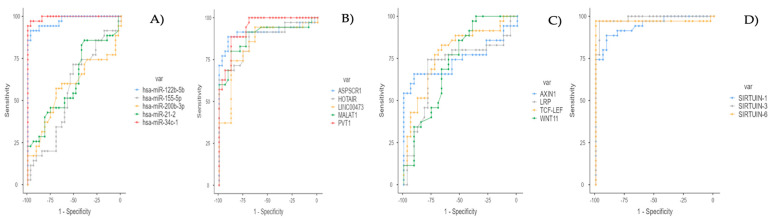
(**A**) ROC curve for miRNA Data. (**B**) ROC curve for lncRNA data (for tests with less than or equal direction). (**C**) ROC curve for mRNA data (for tests with less than or equal direction). (**D**) ROC curve for sirtuin RNA data (for tests with less than or equal direction).

The association between WNT1 and SIRT6 was highly significant in terms of both the original *p*-value (<0.001) and the FDR-adjusted *p*-value (<0.001) (Table 3; Supplementary Tables S1 and S2; Figure 1).

Significant differences were observed in the expression levels of certain biomarkers between the cellular and humoral rejection groups. Notably, hsa-miR-21-2 levels were significantly higher in the cellular rejection group than in the humoral rejection group (31.28 ± 1.45 vs. 29.84 ± 1.29; multivariate analysis: OR = 0.331; 95% CI: 0.126–0.868; *p* = 0.025; adjusted *p* = 0.038).

In addition, hsa-miR-155-5p levels were markedly higher in the cellular rejection group (39.48 ± 1.33 vs. 30.5 ± 1.53), while SIRT-1 levels were significantly lower (25.75 ± 1.72 vs. 28.06 ± 2.56). Multivariate analyses revealed statistical significance for both biomarkers (*p* = 0.018 and *p* = 0.025); although the FDR-adjusted *p*-values were slightly above the threshold (adjusted *p* = 0.054 and 0.052), they support the potential biological relevance of these biomarkers in association with cellular rejection (Table 4).

In comparisons based on the degree of interstitial inflammation, differences were observed in the expression levels of hsa-miR-21-2, TUG, SIRT-1, and TCF-LEF genes, which appeared to correlate with the severity of inflammation (raw *p*-values: 0.023, 0.047, 0.048, and 0.032, respectively). After applying False Discovery Rate (FDR) correction to account for multiple comparisons, the adjusted *p*-values ranged between 0.06 and 0.08. These findings suggest that the expression levels of these genes may be associated with the degree of inflammation and trend toward statistical significance (Table 5).

Table 6 shows statistically significant differences in the expression levels of certain molecular markers in relation to the presence of tubulitis. In univariate analyses, hsa-miR-155-5p levels were significantly increased in patients with tubulitis (raw *p* = 0.032), SIRT-1 expression was significantly decreased (raw *p* = 0.031), and TCF-LEF transcription factor levels were significantly elevated (raw *p* = 0.038). These findings remained statistically significant after correction for multiple testing using the False Discovery Rate (FDR) method: hsa-miR-155-5p (FDR *p* = 0.045), SIRT-1 (FDR *p* = 0.048), and TCF-LEF (FDR *p* = 0.047) (Table 6).

The average values of the TUG gene also showed significant differences between the groups (*p* = 0.034); however, no significant differences were found in the pairwise comparisons. Similarly, the SIRT-6 gene expression levels also showed significant differences according to the glomerulitis groups (*p* = 0.049), but the difference was not significant in the pairwise comparisons between the groups. Additionally, significant differences were found in the mean expression levels of the LRP gene (*p* = 0.003), which was statistically significant between the mild and moderate glomerulitis groups, as well as between the moderate and severe glomerulitis groups. These results suggest that the SNORD61, TUG, SIRT-6, and LRP genes in particular could be biomarkers in the progressive severity levels of glomerulitis (Table 7).

As a result of the analyses, no statistically significant difference was found between many of the genes and molecular markers (Table 8).

IL-6 expression levels tended to increase with the severity of interstitial inflammation (i1: 20.96 ± 1.43 vs. i2: 24.78 ± 2.08; raw *p* = 0.034). However, this increase did not remain statistically significant after multiple testing corrections (FDR and Bonferroni) (adjusted *p* > 0.05). Although a similar upward trend was observed in IL1B gene expression, the difference was not statistically significant (raw *p* = 0.184). These findings suggest an association between elevated IL-6 levels and increasing inflammation severity; however, further studies with larger sample sizes are needed to confirm these results (Table 9) (Figure S1)

In the analysis based on the presence of tubulitis, IL1B levels were found to be significantly elevated, and this difference remained statistically significant after multiple testing correction (FDR) (raw *p* = 0.003; FDR *p* = 0.009). An increase in IL6 levels was also observed in the tubulitis group; although the raw *p*-value was significant, the FDR-adjusted value approached but did not reach statistical significance (raw *p* = 0.046; FDR *p* = 0.069). No significant difference was detected in IFNB1 gene expression (*p* > 0.05) (Table 10) (Figure S2)

## 4. Discussion

This study is one of the first to comprehensively investigate the potential effects of epigenetic regulators—particularly sirtuins, microRNAs, and long non-coding RNAs—on immune response and inflammation in the context of acute kidney transplant rejection. Our findings demonstrate that these molecules are regulated in direct association with the severity of inflammation, and their interactions may play a pivotal role in the rejection process.

The expression levels of SIRT1, SIRT3, and SIRT6 were significantly decreased in patients who developed acute rejection. This suggests that the anti-inflammatory roles of sirtuins are suppressed and that their protective effects are diminished during the rejection process. In particular, SIRT1 inhibits NF-κB pathways and promotes the polarization of macrophages toward the M2 phenotype, thereby exerting an anti-inflammatory effect [7,13]. Our findings indicate that this regulatory effect is weakened during the rejection process [14].

These findings are consistent with the reported immunosuppressive role of SIRT1 in the literature. Indeed, experimental studies using rat renal allograft models have also shown that SIRT1 expression decreases during acute rejection and that this reduction is associated with graft injury [2]. Moreover, T cell-specific SIRT1 deficiency has been shown to suppress the rejection response and prolong allograft survival in murine transplantation models. These data support the immunomodulatory role of SIRT1 in transplantation. Our findings are consistent with these results and demonstrate that SIRT1 is similarly downregulated in human renal transplant tissues [15].

However, conflicting findings have also been reported in the literature. For example, Weng et al. (2024) [16] reported that while the expression levels of several members of the SIRT family change during rejection, SIRT1 levels remain stable. This discrepancy may be due to differences in study design, the timing of tissue sample collection, or biological variability among the models used. Nonetheless, the decrease in SIRT1 observed in our study suggests that this molecule may play a more active role in the rejection process than previously thought, highlighting the need for further research. [16].

In the I2 group, where the severity of interstitial inflammation increased, SIRT1 expression decreased and significant increases were observed in TUG1, hsa-miR-21, and TCF-LEF levels. Similarly, in the T2 group, increased tubulitis severity was associated with decreased SIRT1 levels and increased TCF-LEF and hsa-miR-155 expression. These findings suggest that SIRT1 is downregulated as inflammation progresses, whereas genes involved in regulating inflammatory and fibrotic responses become activated. The elevation of proinflammatory cytokines such as IL-6 and IL-1B reflects an immune activation that parallels these molecular changes.

Among the microRNAs, the increased expression of miR-155 and miR-21 is particularly noteworthy. miR-155 directly targets and suppresses SIRT1, thereby promoting the excessive activation of immune cells [16]. miR-21, on the other hand, is associated with fibrosis and cellular injury and can activate the TGF-β/Smad signaling pathways [17]. The elevation of these microRNAs indicates that both inflammation and fibrotic remodeling are enhanced during the rejection process.

At the lncRNA level, the upregulation of HOTAIR and MALAT1 is consistent with their reported pro-inflammatory and pro-fibrotic effects in the literature [18]. In contrast, the decrease in TUG1 levels suggests that this lncRNA loses its enhancing effect on SIRT1 expression. The TUG1–SIRT1 correlation observed in our study supports this mechanism.

Moreover, the WNT/β-catenin signaling pathway was activated during rejection; this activation was further supported by the increased expression of the TCF-LEF transcription factor. These pathways play a central role in the progression of inflammation and fibrosis, and the literature reports that SIRT6 suppresses these genes.

All these findings suggest that epigenetic regulation plays a decisive role in amplifying the immune response during acute rejection. However, the causal relationships between these molecules have not been fully established. Our findings are hypothesis-generating in nature and highlight the need for advanced in vitro and in vivo studies for mechanistic validation.

In the future, molecules that activate sirtuins, anti-miR strategies targeting miR-155 and miR-21, and antisense approaches against HOTAIR/MALAT1 may serve as promising therapeutic targets for controlling transplant rejection. In particular, therapies aimed at enhancing TUG1 expression could restore SIRT1 levels and suppress inflammation. Additionally, the potential use of these molecules as biomarkers is also worth evaluating.

In conclusion, our study highlights the critical roles of sirtuins and non-coding RNAs in acute kidney transplant rejection, demonstrating their impact on disease progression and their potential as therapeutic targets. Before these findings can be translated into clinical practice, they must be validated through functional and experimental models. Nevertheless, the molecular network we have identified provides a strong foundation for future personalized approaches in both the diagnosis and treatment of acute rejection.

## 5. Conclusions

### 5.1. Molecular Signature Specific to Rejection Type

Our findings suggest that SIRT1 may play a relevant regulatory role in kidney transplant rejection. Although the data presented are observational, they indicate that SIRT1 is not only a potential biomarker but may also be involved in molecular mechanisms underlying rejection. Its downregulation appears to coincide with the activation of Wnt/β-catenin signaling and TCF-LEF transcription factors, as well as with increased miR-21 and miR-155 expression. These molecular alterations are associated with intensified tubulointerstitial inflammation and elevated IL-6 and IL-1β levels.

Such associations imply that both cellular and humoral rejection may be influenced not only by immune-mediated injury but also by epigenetic, transcriptional, and post-transcriptional mechanisms. Notably, in advanced stages of cellular rejection (i2–t2), reduced SIRT1 expression, together with increased levels of miR-21, miR-155, and TCF-LEF, corresponds with enhanced inflammatory responses.

While these findings offer valuable insights, we acknowledge that further validation—particularly through mechanistic in vitro or in vivo studies—is required to confirm the functional relevance of these molecular interactions. Nevertheless, the present results provide an important preliminary framework for future investigations. A deeper understanding of these regulatory networks may ultimately support the development of personalized therapeutic strategies aimed at improving graft outcomes.

### 5.2. Study Limitations and Future Perspectives

Among the limitations of this study are its single-center design and the relatively small sample size, which may reduce the generalizability of the findings. To enhance external validity, further high-level validation studies involving larger and more heterogeneous patient groups are needed. Additionally, it is important to evaluate the molecular findings using high-throughput miRNA profiling techniques and diverse molecular analysis platforms. Confirming the role of SIRT1 and related molecules in different organ transplant models would increase the clinical relevance of this study.

Specifically, future research should elucidate the immunoregulatory effects of miRNAs and lncRNAs in more detail, assessing their potential use as biomarkers or therapeutic targets in transplant rejection. Pharmacologically targeting SIRT1 may offer an innovative strategy for preventing and managing immune-mediated graft injury.

In this study, patients receiving immunosuppressive therapy and those without biopsy samples were excluded. This exclusion was intended to ensure sample homogeneity in molecular analyses and to minimize confounding effects. Immunosuppressive drugs can directly influence gene and RNA expression levels and may mask the intrinsic biological effects of the investigated molecules. Therefore, focusing on a sample not exposed to immunosuppressive treatment is crucial for accurately assessing the independent roles of these molecules in inflammation and rejection. However, this approach may also limit the clinical applicability and generalizability of the study.

Additionally, our study reports various associations between gene and miRNA expression levels and histopathological or clinical findings; however, further studies are needed to perform mechanistic and functional validation experiments. Advanced in vitro and in vivo experimental studies should be conducted to establish causal relationships and to better understand the underlying molecular pathways.

Nevertheless, as a pilot study designed with selective inclusion criteria, the purpose of these preliminary findings is to inform future clinical and translational research. Validating the results in larger and more diverse patient populations is of great importance for enhancing their clinical relevance and applicability.

## Figures and Tables

**Table 1:** Comparison of gender distribution between patient and control groups.

	Groups		Total	Test Statistic	*p*
	Control	Patient			
Gender					
Male	17 (51.5)	25 (71.4)	42 (61.8)	2.071	0.150 ^x^
Female	13 (48.5)	10 (28.6)	23 (38.2)		

Yates’ Correction. ^x^ ki-kare testi (χ^2^).

**Table 2:** Comparison of age, gene expression, and molecular markers between the patient and control groups.

	Control	Patient	Total	Test Statistic	*p*	FDR
SNORD61	29.44 (23.92–32.32)	34.54 (18.73–36.37)	31.63 (18.73–36.37)	147.000	<0.001 ^x^	0.00153
hsa-miR-21-2	29.78 ± 1	30.33 ± 1.5	30.06 ± 1.3	−1.783	0.079 ^y^	0.09085
hsa-miR-34c-1	22.06 ± 1.59	28.65 ± 2.49	25.45 ± 3.92	−13.097	<0.001 ^y^	0.00153
hsa-miR-122b-5b	25.29 (20.18–31.8)	33.96 (27.98–36.63)	30.83 (20.18–36.63)	29.000	<0.001 ^x^	0.00153
hsa-miR-155-5p	29.66 (27.47–33.46)	29.98 (25.65–33.55)	29.88 (25.65–33.55)	511.000	0.418 ^x^	0.41800
hsa-miR-200b-3p	29.94 ± 1	30.43 ± 1.65	30.19 ± 1.38	−1.504	0.138 ^y^	0.14427
GAPDH	22.81 (20.28–27.01)	22.26 (19.12–25.29)	22.4 (19.12–27.01)	701.500	0.129 ^x^	0.14129
MALAT1	22.28 (20.16–27.28)	19.48 (16.96–25.95)	20.57 (16.96–27.28)	1022.500	<0.001 ^x^	0.00153
HOTAIR	22.56 (21.23–26.64)	21.17 (18.66–29.81)	21.53 (18.66–29.81)	1005.500	<0.001 ^x^	0.00153
LINC00473	22.62 (20.63–25.67)	21.22 (17.17–30.53)	21.54 (17.17–30.53)	970.000	<0.001 ^x^	0.00153
TUG	30.82 (28.06–34.44)	27.43 (24.86–33.39)	29.97 (24.86–34.44)	1057.000	<0.001 ^x^	0.00153
Age	35.45 ± 5.39	42.8 ± 13.87	39.24 ± 11.19	−2.909	0.006 ^y^	0.00726
PVT1	25.01 (22.18–28.18)	21.45 (20.57–24.07)	23.15 (20.57–28.18)	1083.500	<0.001 ^x^	0.00153
SIRT-1	29.8 ± 1.37	25.64 ± 2.42	27.66 ± 2.88	8.790	<0.001 ^y^	0.00153
SIRT-3	26.98 ± 1.11	22.77 ± 1.47	24.81 ± 2.49	13.236	<0.001 ^y^	0.00153
SIRT-6	26.74 (24.29–28.8)	22.15 (20.22–28.15)	24.11 (20.22–28.8)	1123.000	<0.001 ^x^	0.00153
WNT1	20.61 (18.5–23.27)	21.54 (19.98–26.47)	21.35 (18.5–26.47)	322.000	0.002 ^x^	0.00287
TCF-LEF	23.71 ± 1.99	25.86 ± 2.25	24.82 ± 2.37	−4.157	<0.001 ^y^	0.00153
LRP	24.16 (21.04–30.82)	25.72 (21.45–28.59)	25.26 (21.04–30.82)	349.000	0.005 ^x^	0.00639
AXIN1	25.9 ± 1.08	27.86 ± 2.41	26.91 ± 2.11	−4.363	<0.001 ^y^	0.00153
IL1B	24.22 (23.14–25.81)	19.83 (16.95–23.2)	23.17 (16.95–25.81)	1149.000	<0.001 ^x^	0.00153
IL6	24.86 ± 0.77	20.73 ± 1.66	22.74 ± 2.45	13.236	<0.001 ^y^	0.00153
IFNB1	24.93 (21.02–27.57)	21.09 (17.23–29.27)	22.88 (17.23–29.27)	1102.000	<0.001 ^x^	0.00153

^x^ Mann–Whitney U test; ^y^ independent samples *t*-test; mean ± standard deviation; median (minimum–maximum).

**Table 3:** Correlation analyses of SIRT-1, SIRT-3, and SIRT-6 expression levels with miRNAs, lncRNAs, Wnt/β-Catenin Pathway, and clinical parameters.

	**SIRT-1**			**SIRT-3**			**SIRT-6**		
	**r**	***p***	**FDR**	**r**	***p***	**FDR**	**r**	***p***	**FDR**
SNORD61	−0.338 ^x^	0.047 ^x^		−0.024 ^x^	0.892 ^x^		−0.223 ^x^	0.198 ^x^	
hsa-miR-21-2	0.151 ^y^	0.387 ^y^		−0.275 ^y^	0.110 ^y^		−0.043 ^x^	0.805 ^x^	
hsa-miR-34c-1	0.056 ^y^	0.749 ^y^		0.062 ^y^	0.725 ^y^		−0.033 ^x^	0.850 ^x^	
hsa-miR-122b-5b	−0.142 ^x^	0.417 ^x^		−0.128 ^x^	0.463 ^x^		−0.064 ^x^	0.715 ^x^	
hsa-miR-155-5p	0.359 ^x^	0.034 ^x^	0.045	0.184 ^x^	0.289 ^x^		0.083 ^x^	0.638 ^x^	
hsa-miR-200b-3p	0.373 ^y^	0.027 ^y^	0.038	0.056 ^y^	0.752 ^y^		0.067 ^x^	0.702 ^x^	
GAPDH	−0.120 ^y^	0.492 ^y^		−0.272 ^y^	0.114 ^y^		−0.165 ^x^	0.343 ^x^	
MALAT1	−0.144 ^x^	0.409 ^x^		0.081 ^x^	0.645 ^x^		−0.179 ^x^	0.303 ^x^	
HOTAIR	−0.412 ^x^	0.014 ^x^	0.025	0.172 ^x^	0.323 ^x^		0.128 ^x^	0.465 ^x^	
LINC00473	−0.207 ^x^	0.233 ^x^		−0.019 ^x^	0.912 ^x^		0.035 ^x^	0.841 ^x^	
TUG	0.154 ^x^	0.377 ^x^		−0.053 ^x^	0.764 ^x^		0.070 ^x^	0.688 ^x^	
PVT1	0.112 ^x^	0.522 ^x^		0.030 ^x^	0.862 ^x^		0.181 ^x^	0.299 ^x^	
GAPDH2	0.289 ^x^	0.093 ^x^		−0.040 ^x^	0.820 ^x^		0.579 ^x^	<0.001 ^x^	
WNT1	0.159 ^x^	0.361 ^x^		0.178 ^x^	0.308 ^x^		0.777 ^x^	<0.001 ^x^	0.001
TCF-LEF	−0.398 ^y^	0.018 ^y^	0.029	0.406 ^y^	0.016 ^y^	0.027	0.294 ^x^	0.086 ^x^	
LRP	0.206 ^x^	0.235 ^x^		0.230 ^x^	0.185 ^x^		0.217 ^x^	0.211 ^x^	
AXIN1	−0.026 ^y^	0.881 ^y^		0.060 ^y^	0.734 ^y^		0.078 ^x^	0.654 ^x^	
IL1B	−0.035 ^y^	0.840 ^y^		−0.073 ^y^	0.678 ^y^		−0.073 ^x^	0.676 ^x^	
IL6	−0.100 ^y^	0.570 ^y^		−0.104 ^y^	0.554 ^y^		−0.003 ^x^	0.987 ^x^	
IFNB1	−0.130 ^x^	0.457 ^x^		−0.029 ^x^	0.868 ^x^		0.296 ^x^	0.085 ^x^	
Creatinine after rejection	0.214 ^x^	0.225 ^x^		−0.073 ^x^	0.682 ^x^		−0.174 ^x^	0.324 ^x^	
Survival Time	−0.105 ^x^	0.550 ^x^		0.167 ^x^	0.339 ^x^		0.249 ^x^	0.150 ^x^	
Age	−0.138 ^y^	0.429 ^y^		−0.315 ^y^	0.066 ^y^		−0.156 ^x^	0.372 ^x^	
Creatinine before rejection	−0.267 ^y^	0.127 ^y^		−0.238 ^y^	0.175 ^y^		−0.027 ^x^	0.881 ^x^	
CRP (mg/L)	0.262 ^x^	0.252 ^x^		−0.122 ^x^	0.598 ^x^		−0.045 ^x^	0.847 ^x^	
Urea (mg/dL)	0.069 ^y^	0.695 ^y^		−0.067 ^y^	0.702 ^y^		−0.329 ^x^	0.054 ^x^	
Na (mmol/L)	0.138 ^x^	0.431 ^x^		−0.065 ^x^	0.709 ^x^		−0.175 ^x^	0.315 ^x^	
K (mmol/L)	0.152 ^x^	0.383 ^x^		−0.417 ^x^	0.013 ^x^		−0.219 ^x^	0.207 ^x^	
Bun (mg/dL)	0.068 ^y^	0.698 ^y^		−0.065 ^y^	0.709 ^y^		−0.327 ^x^	0.055 ^x^	
C4d	0.004 ^x^	0.988 ^x^		0.198 ^x^	0.463 ^x^		0.193 ^x^	0.475 ^x^	

^x^ Spearman’s rho correlation coefficient; ^y^ Pearson correlation coefficient.

**Table 4:** Univariate and multiple binary logistic regression analysis of independent risk factors affecting cellular and humoral rejection types in kidney transplantation patients.

	Rejection Type		Univariate		Multiple		
	Cellular	Humoral	OR (%95 CI)	*p*	OR (%95 CI)	*p*	FDR
SNORD61	32.47 ± 4.18	33.28 ± 4.18	1.048 (0.888–1.237)	0.578	---	---	
hsa-miR-21-2	31.28 ± 1.45	29.84 ± 1.29	0.458 (0.244–0.861)	0.015	0.331 (0.126–0.868)	0.025	0.038
hsa-miR-34c-1	29.26 ± 2.24	28.34 ± 2.61	0.855 (0.637–1.148)	0.297	---	---	
hsa-miR-122b-5b	33.75 ± 1.94	33.55 ± 2.01	0.945 (0.653–1.368)	0.764	---	---	
hsa-miR-155-5p	39.48 ± 1.33	30.5 ± 1.53	1.751 (0.925–3.312)	0.085	3.846 (1.263–11.71)	0.018	0.054
hsa-miR-200b-3p	30.53 ± 1.71	30.38 ± 1.65	0.946 (0.617–1.451)	0.799	---	---	
GAPDH	22.41 ± 1.65	22.12 ± 1.53	0.886 (0.559–1.405)	0.607	---	---	
MALAT1	19.14 ± 1.15	20.06 ± 2.19	1.38 (0.85–2.24)	0.193	---	---	
HOTAIR	20.4 ± 1.34	21.48 ± 2.09	1.746 (0.901–3.381)	0.099	---	---	
LINC00473	21.5 ± 3.12	20.89 ± 1.64	0.883 (0.64–1.218)	0.448	---	---	
TUG	28.2 ± 1.46	27.31 ± 2.13	0.789 (0.545–1.142)	0.209	---	---	
PVT1	21.58 ± 0.93	22.05 ± 1.25	1.475 (0.759–2.865)	0.252	---	---	
GAPDH2	22.18 ± 1.11	22.41 ± 1.73	1.114 (0.684–1.813)	0.665	---	---	
SIRT-1	25.75 ± 1.72	28.06 ± 2.56	0.716 (0.507–1.012)	0.058	0.472 (0.244–0.912)	0.025	0.052
SIRT-3	22.42 ± 1.5	22.95 ± 1.46	1.287 (0.785–2.108)	0.317	---	---	
SIRT-6	21.98 ± 1.19	22.08 ± 1.74	1.043 (0.656–1.659)	0.859	---	---	
WNT1	21.67 ± 1.06	21.85 ± 1.48	1.111 (0.639–1.933)	0.708	---	---	
TCF-LEF	25.32 ± 2.14	26.14 ± 2.3	1.188 (0.854–1.652)	0.306	---	---	
LRP	26.25 ± 2.12	25.4 ± 2.1	0.81 (0.561–1.169)	0.260	---	---	
AXIN1	27.48 ± 2.69	28.05 ± 2.29	1.105 (0.824–1.482)	0.505	---	---	

Mean ± standard deviation; OR: odds ratio; Cox and Snell R^2^ = 48%; Nagelkerke R^2^ = 66.3%.

**Table 5:** Comparison of molecular variants analyzed according to interstitial inflammation severity degree i1–i2 groups in kidney rejection patients.

	i1	i2	Total	Test Statistic	*p*	FDR
SNORD61	34.73 (23.73–36.37)	33.82 (18.73–36.37)	31.63 (18.73–36.37)	72.500	0.425 ^x^	
hsa-miR-21-2	28.34 ± 1.66	31.71 ± 2.69	30.06 ± 1.3	−0.563	0.023 ^y^	0.06
hsa-miR-34c-1	29.26 ± 2.28	30.76 ± 1.65	27.45 ± 3.92	1.439	0.579 ^y^	
hsa-miR-122b-5b	33.84 ± 1.94	33.86 ± 1.24	29.74 ± 5.09	−0.020	0.985 ^y^	
hsa-miR-155-5p	30.15 ± 1.96	30.31 ± 0.67	30.02 ± 1.4	−0.204	0.840 ^y^	
hsa-miR-200b-3p	30.67 ± 1.88	30.19 ± 0.97	30.19 ± 1.38	0.631	0.534 ^y^	
GAPDH	21.84 ± 1.33	22.32 ± 1.88	22.6 ± 1.58	−0.712	0.484 ^y^	
MALAT1	18.86 (16.96–25.24)	19.48 (16.96–25.95)	20.57 (16.96–27.28)	44.000	0.340 ^x^	
HOTAIR	21.22 (18.66–29.81)	21.18 (19.27–22.31)	21.53 (18.66–29.81)	67.500	0.634 ^x^	
LINC00473	21.27 (17.17–30.53)	21.15 (20.15–21.92)	21.54 (17.17–30.53)	75.000	0.340 ^x^	
TUG	26.73 ± 0.94	28.06 ± 2.15	29.2 ± 2.28	2.105	0.047 ^y^	0.08
PVT1	21.07 (20.57–23.98)	21.45 (21.05–23.85)	23.15 (20.57–28.18)	45.000	0.373 ^x^	
GAPDH2	21.79 (20.47–27.6)	22.22 (20.47–23.73)	22.54 (20.03–27.6)	63.000	0.849 ^x^	
SIRT-1	25.68 ± 2.16	22.55 ± 3	24.66 ± 2.88	2.115	0.048 ^y^	0.08
SIRT-3	22.7 ± 1.73	22.66 ± 1.43	24.81 ± 2.49	0.054	0.958 ^y^	
SIRT-6	21.55 (20.43–28.15)	22.15 (20.81–23.64)	24.11 (20.22–28.8)	61.000	0.949 ^x^	
WNT1	21.54 (19.98–26.47)	21.54 (20.26–22.51)	21.35 (18.5–26.47)	61.000	0.949 ^x^	
TCF-LEF	25.2 (20.85–29.09)	29.96 (25.2–31.23)	26.69 (19.25–31.23)	37.500	0.032 ^x^	0.06
LRP	25.54 ± 1.95	24.6 ± 2.54	25.12 ± 2.13	0.975	0.340 ^y^	
AXIN1	27.99 ± 2.7	27.78 ± 2.41	26.91 ± 2.11	0.177	0.861 ^y^	

^x^ Mann–Whitney U Test; ^y^ Independent samples *t*-test; mean ± standard deviation; median (minimum–maximum) i1: inflammation, 10–25% i2: inflammation, 26–50%.

**Table 6:** Comparison of molecular variables analyzed according to tubulitis t1–t2 groups in kidney rejection patients.

	t1	t2	Total	Test Statistic	*p*	FDR
SNORD61	34.03 (18.73–35.59)	35.19 (23.73–36.37)	31.63 (18.73–36.37)	58.000	0.182 ^x^	
hsa-miR-21-2	30.51 ± 1.55	30.42 ± 1.65	30.06 ± 1.3	0.146	0.885 ^y^	0.0900
hsa-miR-34c-1	28.56 ± 2.34	29.28 ± 2.59	25.45 ± 3.92	−0.729	0.473 ^y^	
hsa-miR-122b-5b	33.98 (27.98–36.63)	33.67 (31.63–36.05)	30.83 (20.18–36.63)	92.500	0.749 ^x^	
hsa-miR-155-5p	29.98 (25.65–33.55)	34.65 (29.17–33.2)	31.88 (25.65–33.55)	67.500	0.032 ^x^	0.045
hsa-miR-200b-3p	30.85 ± 1.78	29.62 ± 1.28	30.19 ± 1.38	1.860	0.074 ^y^	
GAPDH	22.09 ± 1.75	22.29 ± 1.35	22.6 ± 1.58	−0.292	0.773 ^y^	
MALAT1	19.48 (16.96–21.67)	19.18 (17.95–25.95)	20.57 (16.96–27.28)	91.000	0.805 ^x^	
HOTAIR	20.78 ± 1.13	21.02 ± 1.54	22.01 ± 1.93	−0.468	0.644 ^y^	
LINC00473	20.72 (17.17–22.35)	21.27 (20.18–30.53)	21.54 (17.17–30.53)	56.500	0.160 ^x^	
TUG	27.91 ± 2	26.83 ± 1.18	29.2 ± 2.28	1.489	0.149 ^y^	0.180
PVT1	21.19 (20.57–23.98)	21.45 (20.57–23.82)	23.15 (20.57–28.18)	81.000	0.844 ^x^	
GAPDH2	22.47 (20.47–27.6)	21.79 (20.47–23.14)	22.54 (20.03–27.6)	102.500	0.416 ^x^	
SIRT-1	26.22 ± 2.35	22.28 ± 2.16	24.66 ± 2.88	11.020	0.031 ^y^	0.048
SIRT-3	22.92 ± 1.6	22.3 ± 1.61	24.81 ± 2.49	0.956	0.348 ^y^	
SIRT-6	22.18 (20.43–28.15)	20.94 (20.43–22.83)	24.11 (20.22–28.8)	114.000	0.168 ^x^	
WNT1	21.72 (19.98–26.47)	21.37 (20.06–22.26)	21.35 (18.5–26.47)	116.000	0.140 ^x^	
TCF-LEF	25.82 ± 2.2	29.06 ± 2.89	26.82 ± 2.37	15.251	0.038 ^y^	0.047
LRP	25.66 ± 2.06	25.67 ± 1.77	25.12 ± 2.13	−0.008	0.994 ^y^	
AXIN1	28.35 (22.33–31.6)	29.65 (24.52–30.85)	26.48 (22.33–31.6)	67.500	0.389 ^x^	

^x^ Mann–Whitney U test; ^y^ independent samples *t*-test; mean ± standard deviation; median (minimum–maximum). t1: mild tubulitis; t2: moderate tubulitis.

**Table 7:** Comparison of molecular variables based on glomerulitis g1-g2-g3 groups in kidney rejection patients.

	g1	g2	g3	Total	Test Statistic	*p*
SNORD61	35.19 (30.73–36.37) ^ab^	35.59 (35.52–36.37) ^a^	32.43 (30.67–34.03) ^b^	31.63 (18.73–36.37)	8.317	0.016 ^x^
hsa-miR-21-2	29.83 ± 1.77	30.79 ± 0.33	30.2 ± 0.87	30.06 ± 1.3	0.536	0.595 ^y^
hsa-miR-34c-1	28.24 ± 2.49	30.48 ± 2.5	28.8 ± 2.09	25.45 ± 3.92	1.029	0.380 ^y^
hsa-miR-122b-5b	33.56 ± 2.35	33.87 ± 0.17	33.47 ± 1.59	29.74 ± 5.09	0.039	0.962 ^y^
hsa-miR-155-5p	29.98 (28.73–30.87)	29.87 (29.39–29.88)	29.88 (25.65–33.55)	29.88 (25.65–33.55)	0.748	0.688 ^x^
hsa-miR-200b-3p	30.19 ± 1.78	29.99 ± 1.24	31.75 ± 2.08	30.19 ± 1.38	1.465	0.260 ^y^
GAPDH	22.22 ± 1.32	23.27 ± 0.91	22.25 ± 1.81	22.6 ± 1.58	0.682	0.520 ^y^
MALAT1	20.46 (17.95–25.95)	19.18 (18.24–20.42)	20.31 (17.95–20.46)	20.57 (16.96–27.28)	2.577	0.276 ^x^
HOTAIR	21.37 (18.66–29.81)	21.22 (19.11–21.62)	21.22 (19.77–21.45)	21.53 (18.66–29.81)	1.263	0.532 ^x^
LINC00473	21.27 (17.17–30.53)	21.15 (20.27–21.82)	20.15 (19.16–21.92)	21.54 (17.17–30.53)	2.229	0.328 ^x^
TUG	26.43 ± 0.91	27.96 ± 0.65	29.37 ± 2.76	29.2 ± 2.28	6.534	0.034 ^y^
PVT1	21.55 ± 0.98	22.17 ± 1.05	22.35 ± 1.12	23.31 ± 1.97	1.221	0.321 ^y^
GAPDH2	21.86 ± 0.96	21.19 ± 0.9	23.82 ± 2.64	22.86 ± 1.79	1.937	0.239 ^y^
SIRT-1	24.65 ± 2.29	23.61 ± 2.62	27.33 ± 2.52	27.66 ± 2.88	2.918	0.083 ^y^
SIRT-3	23.19 (20.22–24.31)	22.48 (19.81–22.49)	24.04 (20.91–26.16)	24.31 (19.81–29.07)	1.839	0.399 ^x^
SIRT-6	21.85 ± 0.92	20.93 ± 0.57	23.76 ± 2.79	24.14 ± 2.54	3.668	0.049 ^y^
WNT1	21.58 (20.06–23.31)	21.37 (19.98–21.39)	21.9 (20.77–26.47)	21.35 (18.5–26.47)	2.797	0.247 ^x^
TCF-LEF	25.96 (20.85–29.15)	24.75 (24.56–31.23)	27.82 (24.92–29.09)	24.69 (19.25–31.23)	0.509	0.775 ^x^
LRP	26.63 ± 1.2 ^a^	23.22 ± 1.81 ^b^	26.6 ± 1.25 ^a^	25.12 ± 2.13	8.604	0.003 ^y^
AXIN1	29.04 ± 2.19	27.97 ± 3.76	28.47 ± 2.15	26.91 ± 2.11	0.265	0.770 ^y^

^x^ Kruskal–Wallis H test; ^y^ One-Way Analysis of Variance (ANOVA); ^a,b^ same letter indicates no difference between the groups; mean ± standard deviation; median (minimum–maximum). g1: less than 25% of glomeruli show leukocyte infiltration and/or endothelial cell swelling in the capillary lumen. g2: 25–75% of glomeruli affected. g3: More than 75% of glomeruli affected.

**Table 8:** Comparison of molecular variables by Ptc1 and Ptc2 groups in peritubular capillaries in kidney transplant rejection patients.

	Ptc1	Ptc2	Total	Test Statistic	*p*
SNORD61	34.03 (23.73–35.59)	34.67 (22.73–36.37)	31.63 (18.73–36.37)	40.000	0.725 ^x^
hsa-miR-21-2	30.01 ± 1.63	30.32 ± 0.81	30.06 ± 1.3	−0.435	0.668 ^y^
hsa-miR-34c-1	28.12 ± 2.14	29.22 ± 2.88	25.45 ± 3.92	−0.970	0.344 ^y^
hsa-miR-122b-5b	33.48 ± 2.23	33.32 ± 0.76	29.74 ± 5.09	0.170	0.867 ^y^
hsa-miR-155-5p	30.39 ± 1.99	30.01 ± 0.5	30.02 ± 1.4	0.454	0.655 ^y^
hsa-miR-200b-3p	30.36 ± 2.14	30.34 ± 0.89	30.19 ± 1.38	0.033	0.974 ^y^
GAPDH	22.15 ± 1.41	23.32 ± 1.7	22.6 ± 1.58	−1.631	0.119 ^y^
MALAT1	19.48 (17.95–25.24)	20.44 (18.67–25.95)	20.57 (16.96–27.28)	28.000	0.198 ^x^
HOTAIR	21.22 (18.66–29.81)	20.78 (19.71–23.32)	21.53 (18.66–29.81)	44.500	1.000 ^x^
LINC00473	21.27 (17.17–25.09)	20.67 (20.15–30.53)	21.54 (17.17–30.53)	51.000	0.668 ^x^
TUG	27.68 ± 2.23	27.14 ± 0.73	29.2 ± 2.28	0.582	0.567 ^y^
PVT1	21.19 (20.57–23.98)	22.33 (21.45–23.15)	23.15 (20.57–28.18)	33.000	0.369 ^x^
GAPDH2	21.79 (20.47–27.6)	21.97 (20.47–23.14)	22.54 (20.03–27.6)	51.500	0.640 ^x^
SIRT-1	25.5 ± 2.47	25.15 ± 2.74	27.66 ± 2.88	0.287	0.777 ^y^
SIRT-3	22.55 ± 1.81	23.39 ± 0.99	24.81 ± 2.49	−1.071	0.297 ^y^
SIRT-6	22.15 (20.43–28.15)	21.79 (20.43–22.69)	24.11 (20.22–28.8)	48.500	0.815 ^x^
WNT1	21.58 (19.98–26.47)	21.59 (20.06–22.26)	21.35 (18.5–26.47)	46.000	0.969 ^x^
TCF-LEF	26.06 ± 2.72	27.33 ± 2.15	24.82 ± 2.37	−1.022	0.320 ^y^
LRP	25.98 ± 1.25	27.13 ± 1.31	25.12 ± 2.13	−1.874	0.076 ^y^
AXIN1	28.02 ± 2.12	28.99 ± 2.07	26.91 ± 2.11	−0.957	0.351 ^y^

^x^ Mann–Whitney U test; ^y^ independent samples *t*-test; mean ± standard deviation; median (minimum–maximum). ptc1: At least 1 or up to 3 peritubular capillaries, each containing 1–4 leukocytes. ptc2: Multiple capillaries, each containing up to 5–10 leukocytes.

**Table 9:** Comparison of IL1B, IL6, and IFNB1 variables based on interstitial inflammation in kidney transplant rejection patients.

	i1	i2	Total	Test Statistic	*p*	FDR
IL1B	20.53 ± 1.77	19.5 ± 1.41	22 ± 2.58	1.371	0.184 ^x^	0.276
IL6	20.96 ± 1.43	24.78 ± 2.08	22.74 ± 2.45	2.011	0.034 ^x^	0.038
IFNB1	21.09 (17.23–22.85)	21.19 (19.61–22.85)	22.88 (17.23–29.27)	52.000	0.656 ^y^	0.656

^x^ Independent samples *t*-test; ^y^ Mann–Whitney U test; mean ± standard deviation; median (minimum–maximum). i1: Inflammation covers 10–25% of the cortex. i2: Inflammation covers 26–50% of the cortex.

**Table 10:** Comparison of IL1B, IL6, and IFNB1 variables based on tubulitis in kidney transplant rejection patients.

	t1	t2	Total	Test Statistic	*p*	FDR
IL1B	19.23 ± 1.31	20.9 ± 1.15	22 ± 2.58	−3.270	0.003 ^x^	0.009
IL6	21.43 ± 1.59	23.09 ± 1.58	22.74 ± 2.45	2.092	0.046 ^x^	0.069
IFNB1	20.79 ± 1.63	21.59 ± 1.09	23.04 ± 2.43	−1.345	0.190 ^x^	0.190

^x^ Independent samples *t*-test; mean ± standard deviation. t1: 1–4 lymphocytes/reservoir (tubule section); t2: 5–10 lymphocytes/reservoir (tubule section).

## Data Availability

Data are available from the corresponding author upon reasonable request.

## Supplementary Materials

The following supporting information can be downloaded at: https://www.mdpi.com/article/10.3390/medicina61061073/s1, Table S1: Comparison of SIRT-1 Expression Levels According to Rejection Type, Gender, Type of Transplant, Interstitial Inflammation, Tubulitis, Glomerulitis, and Peritubular Capillaritis Levels; Table S2: AUC values, cutoff thresholds, and sensitivity/specificity metrics of candidate biomarkers based on ROC analysis; Figure S1: Comparison of IL1B, IL6, and IFNB1 gene expression levels between i1 and i2 groups in patients; Figure S2: Comparison of IL1B, IL6, and IFNB1 gene expression levels between t1 and t2 groups in patients.
