# Supplementary material for: Molecular Crosstalk Between SIRT1, Wnt/β-Catenin Signaling, and Inflammatory Pathways in Renal Transplant Rejection: Role of miRNAs, lncRNAs, IL-1, IL-6, and Tubulointerstitial Inflammation
Source: Medicina (Kaunas). 2025 Jun 11;61(6):1073. doi: 10.3390/medicina61061073 (PMC12195052; doi:10.3390/medicina61061073)

SUPLEMENTER FILE

In the comparison of rejection subtypes, SIRT1 levels were found to be significantly higher in the cellular rejection group (26.75 ± 1.72 vs. 25.06 ± 2.56; p = 0.048, FDR-adjusted p = 0.049). Similarly, patients with high interstitial inflammation scores (i2) had significantly higher levels compared to those with low scores (i1) (28.55 ± 3 vs. 25.68 ± 2.16; p = 0.048, FDR p = 0.050). In patients with high tubulitis scores (t2), significantly higher values were observed compared to those with low scores (t1) (26.22 ± 2.35 vs. 29.28 ± 2.16; p = 0.030, FDR p = 0.046). Sup Table 1

Sup Table - S1 Comparison of SIRT-1 Expression Levels According to Rejection Type, Gender, Type of Transplant, Interstitial Inflammation, Tubulitis, Glomerulitis, and Peritubular Capillaritis Levels

	Mean ± SD	Median (min-max)	Test Statistic	p	FDR
Rejection Type					
Cellular	26,75 ± 1,72	26,65 (23,17 - 29,43)	2,055	0,048[x]	0,049
Humoral	25,06 ± 2,56	25,07 (21,28 - 29,88)			
Sex					
Male	25,42 ± 2,24	25,77 (21,28 - 29,07)	-0,825	0,415[x]	0,498
Female	26,18 ± 2,89	26,23 (21,77 - 29,88)			
Transplant Type					
Living	25,5 ± 2,4	25,82 (21,43 - 29,07)	-0,383	0,704[x]	0,777
Cadaver	25,82 ± 2,52	26,07 (21,28 - 29,88)			
Interstitial Inflammation (i)					
i1	25,68 ± 2,16	26,17 (21,43 - 28,8)	2,115	0,048[x]	0,050
i2	28,55 ± 3	27,07 (22,77 - 28,88)			
Tubulitis (t)					
t1	26,22 ± 2,35	26,17 (21,28 - 29,88)	11,020	0,030[x]	0,046
t2	29,28 ± 2,16	28,07 (21,77 - 29,43)			
Gromerulitis (q)					
g1	23,61 ± 2,62	22,88 (21,43 - 26,52)	2,918	0,083[y]	0,099

g2	27,33 ± 2,52	28,43 (23,17 - 29,43)			
Peritubular Capillaritis (ptc)					
ptc1	25,5 ± 2,47	26,29 (21,28 - 28,8)	0,287	0,777[x]	0,793
ptc2	25,15 ± 2,74	24,68 (22,47 - 29,43)			

[x] Independent Samples T-Test; y One-Way Analysis of Variance (ANOVA). i1: Inflammation covers 10-25% of the cortex. i2: Inflammation covers 26-50% of the cortex. t1: 1-4 cell nuclei in diameter. t2: 5-10 cell nuclei in diameter. g1: Moderate. g2: Severe. ptc1: 1-3 cells. ptc2: 4-6 cells.

Sup Table - S1 continued

	Mean ± SD	Median (min-max)	Test Statistic	p	FDR
Rejection Type					
Cellular	22,42 ± 1,5	22,49 (19,81 - 24,31)	-0,999	0,325[x]	
Humoral	22,95 ± 1,46	23,19 (20,22 - 26,16)			
Sex					
Male	22,85 ± 1,48	23,19 (19,81 - 26,16)	0,540	0,593[x]	
Female	22,55 ± 1,52	22,68 (20,22 - 24,31)			
Transplant Type					
Living	23,06 ± 1,4	23,38 (20,22 - 26,16)	1,374	0,179[x]	
Cadaver	22,38 ± 1,53	22,11 (19,81 - 24,31)			
Interstitial Inflammation (i)					
i1	22,7 ± 1,73	22,87 (19,81 - 26,16)	0,054	0,958[x]	
i2	22,66 ± 1,43	22,48 (20,22 - 24,31)			
Tubulitis (t)					
t1	22,92 ± 1,6	23,44 (19,81 - 26,16)	0,956	0,348[x]	
t2	22,3 ± 1,61	22,48 (20,22 - 24,31)			
Gromerulitis (q)					
g1	22,92 ± 1,46	23,19 (20,22 - 24,31)			
g2	21,59 ± 1,54	22,48 (19,81 - 22,49)	1,839	0,399[y]	
Peritubular Capillaritis (ptc)	23,44 ± 2,06	24,04 (20,91 - 26,16)			
ptc1	22,55 ± 1,81	22,49 (19,81 - 26,16)	-1,071	0,297[x]	

ptc2	23,39 ± 0,99	23,67 (21,91 - 24,31)		

[x] Independent Samples t-Test; [y] Kruskal-Wallis H Test. i1: Inflammation covers 10-25% of the cortex. i2: Inflammation covers 26-50% of the cortex. t1: 1-4 cell nuclei in diameter. t2: 5-10 cell nuclei in diameter. g1: Moderate. g2: Severe. ptc1: 1-3 cells. ptc2: 4-6 cells.

Sup Table - S1 continued

	Mean ± SD	Median (min-max)	Test Statistic	p
Rejection Type				
Cellular	21,98 ± 1,19	22,23 (20,43 - 23,93)	143,500	0,862[x]
Humoral	22,08 ± 1,74	21,55 (20,22 - 28,15)		
Sex				
Male	22,09 ± 1,74	22,15 (20,22 - 28,15)	120,000	0,869[x]
Female	21,95 ± 1,05	21,82 (20,72 - 23,64)		
Transplant Type				
Living	22,36 ± 1,81	22,25 (20,22 - 28,15)	193,500	0,151[x]
Cadaver	21,64 ± 1,06	21,43 (20,43 - 23,64)		
Interstitial Inflammation (i)				
i1	22,22 ± 1,91	21,55 (20,43 - 28,15)	61,000	0,949[x]
i2	21,91 ± 0,97	22,15 (20,81 - 23,64)		
Tubulitis (t)				
t1	22,4 ± 1,84	22,18 (20,43 - 28,15)	114,000	0,168[x]
t2	21,33 ± 0,86	20,94 (20,43 - 22,83)		
Gromerulitis (q)				
g1	21,85 ± 0,92	22,15 (20,43 - 23,29)	3,668	0,049[y]
g2	20,93 ± 0,57	20,81 (20,43 - 21,55)		
Peritubular Capillaritis (ptc)	23,76 ± 2,79	23,87 (20,72 - 28,15)		
ptc1	22,22 ± 2,01	22,15 (20,43 - 28,15)	48,500	0,815[x]
ptc2	21,69 ± 0,95	21,79 (20,43 - 22,69)		

[x] Independent Samples T-Test; y One-Way Analysis of Variance (ANOVA). i1: Inflammation covers 10-25% of the cortex. i2: Inflammation covers 26-50% of the cortex. t1: 1-4 cell nuclei in diameter. t2: 5-10 cell nuclei in diameter. g1: Moderate. g2: Severe. ptc1: 1-3 cells. ptc2: 4-6 cells.

Sup Table – S2 AUC values, cutoff thresholds, and sensitivity/specificity metrics of candidate biomarkers based on ROC analysis.

	Cut-off value	AUC (%95 CI)	p	Sensitivity (%)	Specificity (%)	PPV (%)	NPV (%)
SNORD61	≥32,43	0,873 (0,768 - 0,978)	<0,001	80%	100%	100%	82,50%
hsa-miR-21-2	---	0,617 (0,483 - 0,752)	0,096	---	---	---	---

hsa-miR-34c-1	≥24,84	0,995 (0,984 - 1)	<0,001	94,29%	100%	100%	94,29%
hsa-miR-122b-5b	≥30,95	0,975 (0,944 - 1)	<0,001	91,43%	96,97%	96,97%	91,43%
hsa-miR-155-5p	---	0,558 (0,418 - 0,697)	0,414	---	---	---	---
hsa-miR-200b-3p	---	0,58 (0,44 - 0,719)	0,259	---	---	---	---
GAPDH	---	0,393 (0,257 - 0,528)	0,128	---	---	---	---
MALAT1	≤20,46	0,885 (0,801 - 0,969)	<0,001	80%	87,88%	87,50%	80,56%
HOTAIR	≤21,22	0,871 (0,784 - 0,957)	<0,001	62,86%	100%	100%	71,74%
LINC00473	≤21,39	0,84 (0,741 - 0,938)	<0,001	74,29%	87,88%	86,67%	76,32%
TUG	≤29,95	0,915 (0,837 - 0,993)	<0,001	88,57%	90,91%	91,18%	88,24%
PVT1	≤23,15	0,938 (0,886 - 0,99)	<0,001	88,57%	87,88%	88,57%	87,88%
GAPDH	≤23,82	0,697 (0,565 - 0,829)	0,005	91,43%	54,55%	68,09%	85,71%
SIRT-1	≤28,56	0,947 (0,898 - 0,996)	<0,001	88,57%	90,91%	91,18%	88,24%
SIRT-3	≤24,31	0,986 (0,965 - 1)	<0,001	97,14%	96,97%	97,14%	96,97%
SIRT-6	≤23,93	0,972 (0,919 - 1)	<0,001	97,14%	100%	100%	97,06%
WNT1	≥20,94	0,721 (0,599 - 0,844)	0,002	77,14%	60,61%	67,50%	71,43%
TCF-LEF	≥24,56	0,781 (0,67 - 0,893)	<0,001	77,14%	72,73%	75,00%	75,00%
LRP	≥25,3	0,698 (0,566 - 0,83)	0,005	74,29%	78,79%	78,79%	74,29%
AXIN1	≥27,61	0,745 (0,62 - 0,87)	0,001	65,71%	90,91%	88,46%	71,43%

Sup figure S1. Comparison of IL1B, IL6, and IFNB1 gene expression levels between i1 and i2 groups in patients.

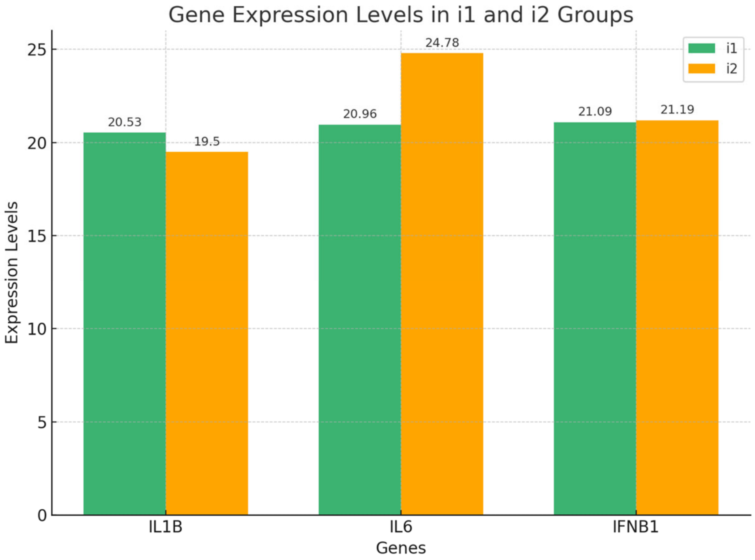

Sup figure S2 . Comparison of IL1B, IL6, and IFNB1 gene expression levels between t1 and t2 groups in patients.

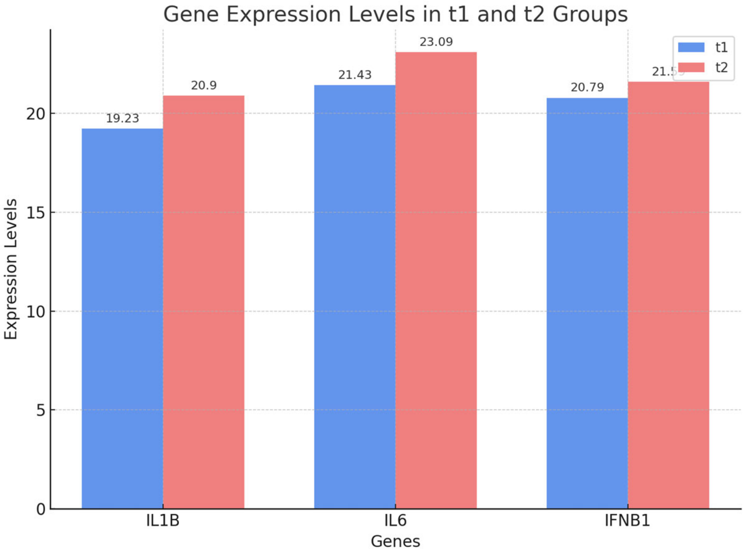

Supplement: Supplementary file 1 [file medicina-61-01073-s001.zip › medicina-3644554-supplementary.pdf]
